# Effect of co-twin gender on neurodevelopmental symptoms: a twin register study

**DOI:** 10.1186/s13229-016-0074-z

**Published:** 2016-01-19

**Authors:** Jonna Maria Eriksson, Sebastian Lundström, Paul Lichtenstein, Susanne Bejerot, Elias Eriksson

**Affiliations:** Department of Clinical Neuroscience, Karolinska Institutet, KIND, Gävleg 22, SE-113 30, Stockholm, Sweden; Centre for Ethics, Law and Mental Health (CELAM), University of Gothenburg, Gothenburg, Sweden; Gillberg Neuropsychiatry Centre, University of Gothenburg, Gothenburg, Sweden; Department of Medical Epidemiology and Biostatistics, Karolinska Institutet, Stockholm, Sweden; School of Medical Sciences, Örebro University, Örebro, Sweden; Department of Pharmacology, Institute of Neuroscience and Physiology, Sahlgrenska Academy, University of Gothenburg, Gothenburg, Sweden

**Keywords:** Twin study, Autistic disorder, Asperger syndrome, Attention-deficit hyperactivity disorders, Symptom assessment

## Abstract

**Background:**

Autism spectrum disorder (ASD) and attention-deficit/hyperactivity disorder (ADHD) are neurodevelopmental disorders thought to have both genetic and environmental causes. It has been hypothesized that exposure to elevated levels of prenatal testosterone is associated with elevated traits of ASD and ADHD. Assuming that testosterone levels from a dizygotic male twin fetus may lead to enhanced testosterone exposure of its co-twins, we aimed to test the prenatal testosterone hypothesis by comparing same-sex with opposite-sex dizygotic twins with respect to neurodevelopmental symptoms.

**Methods:**

Neuropsychiatric traits were assessed in a population-based twin cohort from the Child and Adolescent Twin Study in Sweden (CATSS). Parental interviews were conducted for 16,312 dizygotic twins, 9 and 12 years old, with the Autism—Tics, ADHD, and other Comorbidities inventory (A-TAC).

**Results:**

Girls with a female co-twin had an increased risk of reaching the cut-off score for ADHD compared with girls with a male co-twin. Both boys and girls with a female co-twin displayed a larger number of traits related to attention deficit and repetitive and stereotyped behaviors than those with a male twin. In girls, this also extended to social interaction and the combined measures for ASD and ADHD, however, with small effect sizes.

**Conclusions:**

Our results are reverse to what would have been expected from the prenatal testosterone hypothesis but consistent with a previous study of ASD and ADHD traits in dizygotic twins. The seemingly protective effect for girls of having a twin brother may be an effect of parent report bias, but may also be an unexpected effect of sharing the intrauterine environment with a male co-twin.

## Background

Autism spectrum disorder (ASD) and attention-deficit/hyperactivity disorder (ADHD) are neurodevelopmental disorders that are more commonly diagnosed in males than in females. The heritability is high in both ASD [1] and ADHD [2], but no individual gene variants exerting a major impact on the susceptibility have as yet been identified [1, 2]. Further, ASD and ADHD are frequently co-occurring, implying some shared etiology [3]. There is some evidence that dizygotic twins are more concordant for ASD than non-twin siblings, indicating that also intrauterine factors may be involved [4].

Several possible explanations for the high male-to-female ratio in autism have been put forward. The sex-differential threshold liability model for ASD proposes that a greater genetic burden is required for females than for males to develop ASD, implicating the existence of a yet unidentified female protective effect [5–7]. The extreme male brain theory of autism, on the other hand, describes an autistic personality characterized by extremes of typical male personality traits [8] in terms of systemizing skills and weaknesses in empathy [9]. This connection to sex-related differences might be one clue to the etiologies of ASD, and it has been hypothesized that exposure to elevated levels of prenatal testosterone is associated with more autistic traits and thus also may be a risk factor for developing ASD. This has been supported by studies of the association between autistic traits and testosterone levels in amniotic fluid [10–12].

Testosterone passes the blood–brain barrier and binds to the androgen receptor in the brain in both males and females, and a permanent (organizational) impact of prenatal testosterone exposure on fetal neurodevelopment has been confirmed in animal studies [13]. The higher testosterone levels in male fetuses are reflected by considerably higher testosterone levels also in the amniotic fluid compared with those observed for female fetuses [14]. Studies on rodents have shown that testosterone from male fetuses transfer to adjacent fetuses via amniotic diffusion [15], and this phenomenon has been proposed to occur also in human twin pregnancies [16]. In this vein, studies of prenatal testosterone transfer in humans suggest that having a male co-twin may masculinize various aspects of the phenotype in women, including body mass index [17], tooth crown size [18], the eruption of teeth [19], the second- to fourth-digit ratio [20], leukocyte telomere length [21], and cerebral lateralization [22]. Some reports suggest that a corresponding virilization may take place also with respect to behavioral parameters, such as aggression [23] and sensation seeking [16, 24]; however, most of these studies have been based on small samples, and they are to some extent contradicted by reports failing to detect clear-cut behavioral differences between same-sex and opposite-sex female twins [25–29]. Similarly controversial is the possible influence of having a male twin for the risk of developing disordered eating in females, some studies suggesting a protecting effect [30, 31] while others have failed to replicate this finding [32–34].

Assuming that having a male co-twin increases the prenatal exposure to testosterone, the prenatal androgenization theory of autism [10] would predict a higher rate of autistic traits in females with a male co-twin. Likewise, since also ADHD is more prevalent in males than females, and since it has been suggested that prenatal testosterone exposure of girls increases the risk also for ADHD [35], ADHD traits could also be expected to be enhanced in opposite-sex female twins. A previous study, comparing neuropsychiatric traits in dizygotic twins with either a male or a female co-twin, however, revealed the counter-intuitive result that female index twins with a male co-twin had a lower rate of parent-reported ADHD- and ASD-related traits than those with a female co-twin [36]. In the present study, we have addressed the same issue by analyzing data obtained within the Child and Adolescent Twin Study in Sweden (CATSS) [37] on parent-reported symptoms of ADHD and ASD in dizygotic twins having either a male or female co-twin.

## Methods

### Participants

This study was conducted using data from CATSS, an ongoing, population-based longitudinal twin study targeting all twins born in Sweden since July 1, 1992, at 9 or 12 years of age. The CATSS has a response rate of 80 % with little influence of the presence of neurodevelopmental disorders on the response rate [37]. Zygosity was determined by a DNA test [38]. A total of 8156 dizygotic twin pairs (4219 male–female, 1808 female–female, 2129 male–male) were included in the present study.

### The Autism—Tics, AD/HD, and other Comorbidities inventory

ADHD and autistic traits were assessed with the Autism—Tics, AD/HD, and other Comorbidities inventory (A-TAC) [39–41] which is a parental telephone interview for assessing neuropsychiatric problems in children. All interviews were conducted by professional interviewers who had undergone a brief introduction to psychiatry and twin research. A-TAC comprises 96 questions organized in 20 modules regarding clinically meaningful areas of psychiatric or psychological problems. The ASD domain comprises three modules: “language,” “social interaction,” and “flexibility” (mirroring the DSM-IV subdomain of “restricted repetitive and stereotyped patterns”). The AD/HD domain comprises the modules “impulsiveness/activity” and “concentration/attention.” The remaining 15 modules cover symptoms on other neuropsychiatric problems with less specific relation to ASD or ADHD in the DSM-IV. Response options for each item are “yes” (1), “yes, to some extent” (0.5), and “no” (0). Seventeen items were used to assess behavior representing the different ASD criteria defined by the DSM-IV [37]. The A-TAC domain and module scores represent the summed scores of the included items. The internal consistency has been shown to be good for the 17 ASD items (*α* = 0.86) and excellent for the 19 AD/HD items (*α* = 0.90) [37]. The present study used the cut-offs for ASD and ADHD caseness from the A-TAC validation study [40]. Two cut-offs for ASD were used: a low cut-off of ≥4.5 points, which yielded a sensitivity of 0.96 and specificity of 0.88, and a high cut-off of ≥8.5 points (sensitivity 0.71, specificity 0.95). For AD/HD, a low cut-off of ≥6 yielding a sensitivity of 0.98 and specificity of 0.81, and a high cut-off of ≥12.5 (sensitivity 0.52, specificity 0.95) were used.

### Statistical analysis

We studied differences in neuropsychiatric traits, as measured by the A-TAC modules, in index twins with either a female or male co-twin. In the main analysis, possible differences in scores for the composite modules for ASD and AD/HD were analyzed. In sensitivity analyses, differences with respect to cut-off rates for these modules, as well as differences with respect to scores of individual ASD and AD/HD submodules, were assessed. The three remaining A-TAC single modules spanning at least 5 points or more in the cohort were also tested to serve as control variables. In same-sex pairs, the index twin was selected randomly, in opposite-sex pairs the twin of the analyzed sex was selected as index twin. Due to non-normality and heterogeneity of variances of the residual distributions, we used Mann–Whitney *U* test to analyze differences in module scores between index twins with a co-twin brother and index twins with a co-twin sister. The chi-square test was used to compare proportions of above cut-off scores between groups. In all analyses, both twins were excluded if either twin had a missing module score (46 pairs (0.6 %) for ASD score and 65 pairs (0.8 %) for ADHD score). Quade’s rank analysis of covariance [42] was used to analyze interaction effects. Since the main analysis comprises only two comparisons, and the other comparisons are to be regarded as sensitivity analyses, no correction of *p* values for multiple comparisons was deemed necessary.

### Ethics statement

Participants in the CATSS study are protected by informed consent process—they are informed of what is being collected and given the option to withdraw their consent and discontinue participation. The CATSS-9/12 study has ethical approval from the Karolinska Institute Ethical Review Board: Dnr 02-289 and 2010/507-31/1.

## Results

### Distribution of scores

As could be expected, the score of the outcome variables were extremely skewed. A total of 48 % of the male twins and 58 % of the female twins scored 0 points on autistic traits (Table 1). The female index twins with a female co-twin scored higher than the female index twins with a male co-twin on both autistic traits and ADHD traits (Table 2) but with small effects (*r* < 0.1). There was no significant interaction effect between co-twin sex and age on autistic traits or autistic traits. Neither was there any interaction between co-twin sex and ADHD traits on autistic traits nor between co-twin sex and autistic traits on ADHD traits.

**Table 1:** Distribution of scores (%) for index twins with either a female or male co-twin

	ADHD				ASD			
	FF	FM	MF	MM	FF	FM	MF	MM
0	45.1	47.4	34.2	35.5	55.7	60.6	47.7	48.2
0.5	11.3	11.6	10.0	10.0	17.8	16.7	17.2	17.5
1	8.2	8.9	7.6	8.2	11.1	9.3	12.7	11.5
1.5	6.3	6.1	5.9	6.1	4.8	4.6	6.0	6.2
2	4.8	5.4	5.7	4.7	3.4	2.8	4.8	4.8
2.5	3.2	3.7	4.9	4.8	1.5	1.5	2.3	2.8
3	3.4	3.1	4.0	3.9	1.6	1.2	2.0	1.7
3.5	3.1	2.2	3.5	3.1	0.9	0.6	1.1	1.6
4	2.3	2.0	2.7	2.9	0.6	0.6	0.9	0.9
4.5–5	2.9	2.7	5.4	5.5	0.9	0.5	1.7	1.2
5.5–6	2.5	2.3	3.7	3.6	0.4	0.4	0.9	0.8
6.5–7	1.7	1.2	2.9	2.7	0.3	0.3	0.7	0.8
7.5–8	1.0	0.7	1.9	1.5	0.2	0.1	0.6	0.4
>8	4.0	2.7	7.5	6.9	0.5	0.6	1.4	1.4

ADHD and ASD scores from the Autism—Tics, AD/HD, and other Comorbidities inventory (A-TAC)

*FF* female with a female co-twin, *FM* female with a male co-twin, *MF* male with female co-twin, *MM* male with a male co-twin

**Table 2:** Differences in autistic and ADHD traits between index twins with either a female or a male co-twin

		Female co-twin		Male co-twin				
	Index twin	*N*	Mean rank	*N*	Mean rank	Mann–Whitney *U*	*P*	*r*
ADHD traits	Female	1793	3063	4195	2961	3,626,673	0.03	0.03
	Male	4190	3179	2109	3091	4,295,000	0.06	0.02
Autistic traits	Female	1795	3099	4197	2953	3,582,249	0.001	0.04
	Male	4197	3160	2115	3150	4,425,446	0.8	–

ADHD and autistic traits measured by the Autism—Tics, AD/HD, and other Comorbidities inventory (A-TAC). *r* effect size

### Exploring the single modules

To further explore the results above, the differences in rank scores were also tested in each of the A-TAC modules spanning at least 5 points in scores: all differences pointed to a higher rank sum in the index twins with a female co-twin. Among the girls, the index twins with a female co-twin had higher rank scores than those with a male co-twin in four modules: “perception” (*U* = 3,664,254, *p* = 0.004, *r* = 0.04), concentration/attention (*U* = 3,605,112, *p* = 0.001, *r* = 0.04), social interaction (*U* = 3,659,944, *p* = 0.01, *r* = 0.03), and flexibility (*U* = 3,677,626, *p* = 0.005, *r* = 0.04). In boys, twins with a female co-twin had higher rank scores on concentration /attention (*U* = 4,261,827, *p* = 0.003, *r* = 0.04) and flexibility (*U* = 4,368,327, *p* = 0.006, *r* = 0.03) than those with a male co-twin. No differences were found in the modules language, impulsiveness/activity, “emotion,” or “opposition.”

### Differences in cut-off scores for ADHD and ASD

The pattern of proportions of twins with either a male or female co-twin reaching the cut-off scores for ADHD and ASD were studied (Table 3). A higher frequency of index twins with a female co-twin that reached the low cut-off score for ADHD could be seen in the females. This also extended to the combined sample of males and females, *χ*^2^ (1, *N* = 8,091) = 11.1, *p* = 0.001, *ϕ* = 0.04, with odds ratio 1.27 (95 % CI = 1.10 to 1.47). Although the frequencies were consistently higher in the twins with a female co-twin also for the ASD cut-off scores, in both girls and boys, these differences did not reach statistical significance (Table 3).

**Table 3:** Number of children, *n* (%), with scores for ADHD and autistic traits above validated cut-offs

	ADHD				ASD			
Cut-off	≥6		≥12.5		≥4.5		≥8.5	
Index twin	F**	M	F	M	F	M	F	M
Female co-twin	137 (7.6)	595 (14.2)	17 (0.9)	125 (3)	40 (2.2)	220 (5.2)	10 (0.6)	58 (1.4)
Male co-twin	247 (5.9)	274 (13)	39 (0.9)	52 (2.5)	81 (1.9)	103 (4.9)	23 (0.5)	25 (1.2)

ADHD and autistic traits measured by the Autism—Tics, AD/HD, and other Comorbidities inventory (A-TAC)

*F* female index twin, *M* male index twin

**p = 0.01 for *χ* ^2^ analysis between female and male co-twins

## Discussion

We have studied the effect of having a female or male co-twin on traits for autism and ADHD in a cohort of 9 and 12 year old Swedish dizygotic twins. Our study aimed to explore the hypothesis that elevated levels of prenatal testosterone increase the risk of developing autistic and/or ADHD-related traits, under the assumption that elevated testosterone levels from a dizygotic male twin fetus will increase testosterone exposure of its co-twin, leading to a masculinization of the brain. Contrary to what we had predicted, no increase in autistic traits could be found in girls with a male co-twin compared with those with a female co-twin. Instead, the opposite result was found, that girls with a female co-twin were reported to have more ADHD and autistic traits than girls with a male co-twin. It was also significantly more common for boys and girls with a female co-twin to reach the lower cut-off for ADHD than for those with a male co-twin. Although the same pattern was notable with respect to the cut-off for ASD, these differences were too small to reach statistical significance. In the analysis of the different A-TAC module scores, differences in scores between having a female co-twin and a male co-twin were present for the AD/HD module attention/concentration, the ASD modules flexibility, and social interaction, and also for the perception module. Although the perception module, which includes items regarding sensory hyper reactivity, is a stand-alone module in the A-TAC, it should be noted that the DSM-5, in addition to the DSM-IV criteria, includes hyper-/hypo-reactivity to sensory input in its ASD criteria. Thus, the difference found in the perception can also be interpreted as related to ASD. Notably, all module score differences for boys and girls alike pointed in the same direction, that is, towards higher scores in twins with a female co-twin.

Although the main analysis included two comparisons, no correction of *p* values was undertaken. Whereas the association between sex of co-twin and ASD scores would remain significant also following such an adjustment, the association between sex of co-twin and ADHD scores would not. The latter relationship, not being a chance finding, however, gains support from the analysis regarding cut-off for ADHD diagnosis, as well as from the fact that our observation is in line with a previous report from Attermann and co-workers [36]; these authors, however, found no corresponding effect for males. Also, a greater aggregation of autistic symptoms in siblings of female probands with high scores as compared to siblings with male probands with high scores, interpreted as a protective effect exerted by the female sex, has previously been reported on a study partly based on the same cohort [6].

There are several possible explanations for the higher scores in female twins with a twin sister, and they may not be the same for the ASD and ADHD traits. One explanation could be parent rater contrast effects, that is, an existing difference between the twins amplified by the rater. As the rated traits of ASD and ADHD are generally more frequent in males, the risk of an underreporting of traits in the female twin due to a contrast effect will be larger in the opposite-sex twins than in the same-sex twins. Additionally, the contrast effect may result in underreporting also when the co-twin is afflicted by a neuropsychiatric disorder, and as both ASD and ADHD are more common in boys than girls, such an effect would have a bigger impact on the twins with a male co-twin. Such a bias playing a role gains support from the fact that we, unlike Attermann and co-workers [36], observed a corresponding effect of the sex of the co-twin on the attention/concentration and flexibility modules also in male twins. On the other hand, one should note that no such effect was seen for traits of impulsiveness/activity, language, emotion, and opposition in either sex, similarly known to differ between genders. Moreover, previous studies of parent rating contrast effect in dizygotic twins have shown larger effects in straightforward traits as shyness and hyperactivity [43, 44], while a less observable trait such as attention has shown very little contrast effect in this age group [45]. In addition to the possible bias from twin comparison, underreporting may also arise from concealed traits. Thus, as girls are generally more socially responsive [46] and able to recognize need-of-help [47], there is a possibility that, in cases where both twins have many ADHD symptoms, the girl makes a greater effort to manage herself in order to take responsibility also for her co-twin. Thus, the severity of ADHD symptoms in a girl with a co-twin with even more traits might be underestimated.

Although this study does not provide support for the prenatal testosterone hypothesis for autism, it also does not refute it. First, both the reporting bias discussed above (see also [36]), and the tentative protective effect of the female gender [5–7], may serve to mask a risk-enhancing effect of increased in utero testosterone exposure in females with a male twin. Second, the functional relevance effect of testosterone transfer between human twins is not fully established. Third, the risk associated with high prenatal testosterone levels may be associated with testosterone levels that are higher than those tentatively caused by amniotic testosterone transfer in humans. And fourth, one cannot exclude the existence of a complex association between prenatal testosterone and neuropsychiatric symptoms, where moderately elevated levels (caused by diffusion from a male co-twin) may lead to a reduced responsiveness to the hormone, hence reducing the influence of a sudden rise in testosterone level produced by the fetus or the mother.

There are some limitations to the present study. One is that neuropsychiatric traits were only assessed by parental assessment and by means of layman interviews. However, A-TAC is thoroughly validated and the cut-offs reliably mirror current estimates of the prevalence of ADHD and ASD, respectively [48], in the Swedish population. Another limitation is that the DSM-IV criteria for ASD, on which A-TAC is based, mainly reflect the symptomatology displayed by males and that the presentation of ASD may be different in girls [49], hence making the A-TAC assessment of autistic traits in girls less certain. The major strengths of this study are that it is based on a large sample of twins and the use of a well-established and fairly comprehensive rating scale.

## Conclusions

There is a small but increased risk for dizygotic twins with a female co-twin of displaying a high degree of parent-reported ADHD traits when compared with those with a male co-twin, indicating a possible protective effect of having a twin brother. Having a female co-twin is also associated with slightly elevated autistic traits, especially in the domains concerning flexibility of thought and social interaction, thus adding an intriguing complication to the hypothesis that elevated prenatal testosterone levels causes an increased frequency of autistic traits.

## Abbreviations

ADHD: attention-deficit/hyperactivity disorder
ASD: autism spectrum disorder
A-TAC: Autism—Tics, ADHD, and other Comorbidities inventory
CATSS: Child and Adolescent Twin Study in Sweden
